# Spontaneous Phase Transfer-Mediated Selective Removal of Heavy Metal Ions Using Biocompatible Oleic Acid

**DOI:** 10.1038/s41598-017-17092-9

**Published:** 2017-12-01

**Authors:** Jeehan Chang, Sooyeon Yoo, Wooju Lee, Dongchoul Kim, Taewook Kang

**Affiliations:** 1 Department of Chemical and Biomolecular Engineering, Sogang University, Seoul, 04107 Korea; 2 Department of Mechanical Engineering, Sogang University, Seoul, 04107 Korea

## Abstract

Here, we propose an environmentally benign removal technique for heavy metal ions based on selective and spontaneous transfer to oleic acid. The ions can be removed *via* (1) the selective and rapid complexation with the carboxylic end of oleic acid at an oleic acid/water interface, and (2) the diffusion of such complex into the oleic acid layer. A wide variety of heavy metal ions such as Cu^2+^, Pb^2+^, Zn^2+^, and Ni^2+^ can be selectively removed over K^+^ and Na^+^. For example, the concentration of Cu^2+^ is reduced to below 1.3 ppm within 24 h, which corresponds to the level of Cu^2+^ permitted by the Environmental Protection Agency. The addition of ethylenediamine ligand to the metal ion solutions is also shown to enhance the phase transfer. The removal efficiency is increased by up to 6 times when compared with that in the absence of the ligand and follows the order, Cu^2+^ (99%) > Pb^2+^ (96%) > Zn^2+^ (95%) > Ni^2+^ (65%). Moreover, the removal time can be shortened from 24 h to 1 h. The effect of an emulsion induced by a mechanical agitation on the removal of heavy metal ion is also studied.

## Introduction

A few years ago, accidental lead (Pb^2+^) poisoning in drinking water in the city of Flint, Michigan, caused many people, especially children younger than 5 years, to suffer from skin rashes, as well as cognitive and behavioral disorders^1^. This incident serves to demonstrate that the removal of heavy metal ions is still one of critical issues in modern public health^2,3^.

To date, the removal of heavy metal ions in water has been performed by a variety of techniques^4–9^. One of the most popular techniques for this purpose is chemical precipitation due to its operational simplicity and low cost^10,11^. Precipitation method utilizes basic precipitants such as NaOH and Ca(OH)_2_ in order to produce insoluble metal salts^12^. However, after precipitation, an acid neutralization process is required to lower the pH of the solution. Adsorption is an alternative technique to chemical precipitation^13,14^. Porous materials such as activated carbon, mesoporous oxide and zeolite are widely used as adsorbents after surface modification^15–21^. Carbon nanotubes have been also tested as adsorbents, but they have several issues such as potential toxicity and mass production to be addressed for fieldwork applications^22–24^.

Here, we report a facile method for the removal of heavy metal ions by taking advantage of spontaneous and selective phase transfer into an oil phase. The phase transfer is driven by interfacial complexation and diffusion. Biocompatible oleic acid is chosen as the oil phase since its carboxylic end is known to form stable complexes with many heavy metal ions^25,26^. Our proposed method has several key advantages over chemical precipitation and adsorption. First, it requires neither specially-designed adsorbent nor toxic precipitant. Second, oleic acid is one of lipids and is known to be biocompatible^27,28^. Lastly, oleic acid which captures aqueous heavy metal ions is quite easy to be separated from water due to the density difference between water and oleic acid. Water and oleic acid are immiscible. Therefore, our method does not require an additional filtration to remove insoluble precipitates or fine solid adsorbents.

## Results and Discussion

**Figure 1:**
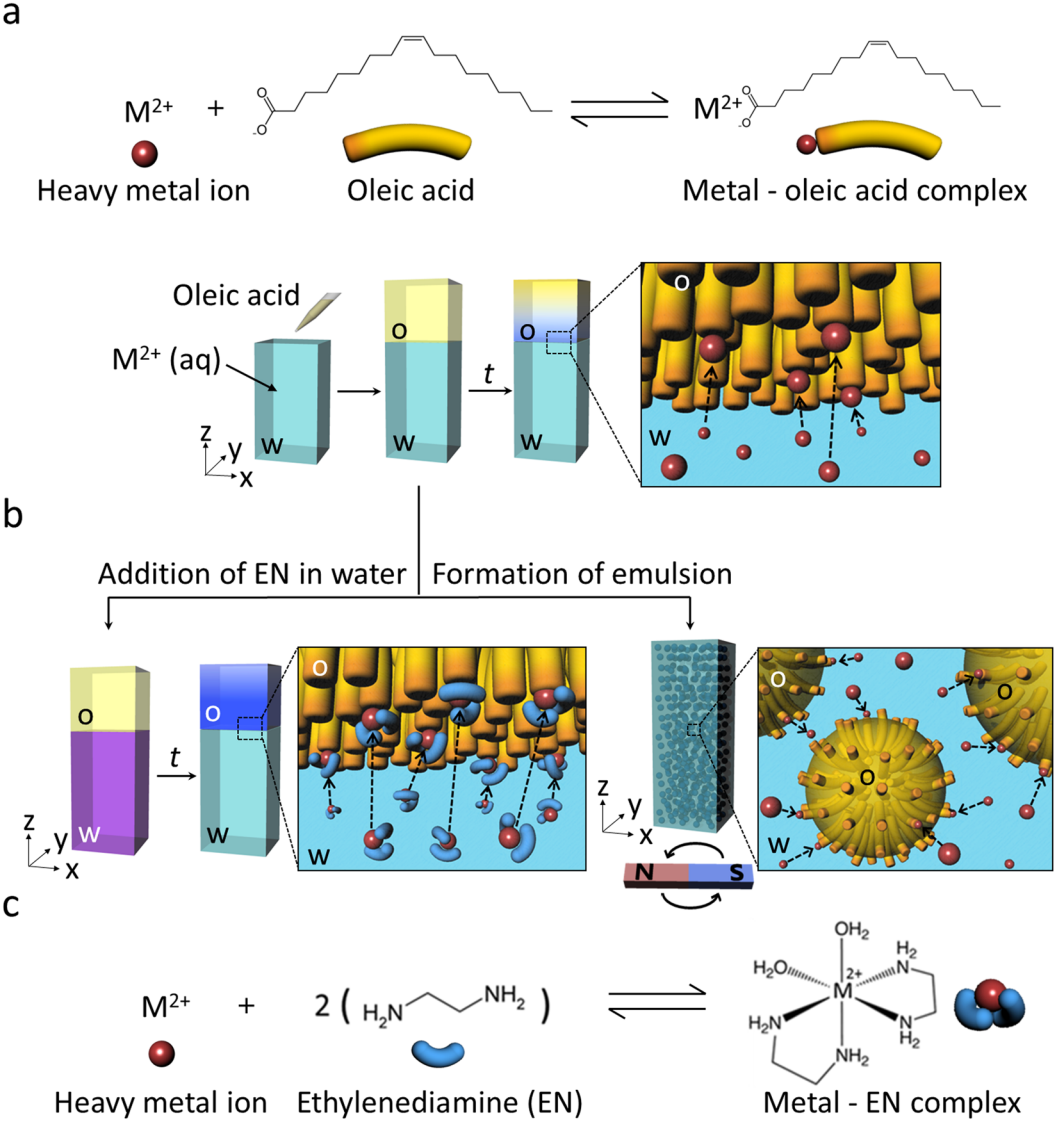
Schematic illustration of the spontaneous phase transfer-mediated selective removal of heavy metal ions using oleic acid. (**a**) Complex formation between heavy metal ions and the carboxylic end of oleic acid (top) and experimental procedure for the removal of heavy metal ions by using oleic acid (bottom). (**b**) Chemical and mechanical enhancements for heavy metal ions removal. (**c**) Complex formation of heavy metal ions with ethylenediamine ligand.

Our idea for the removal of heavy metal ions is schematically illustrated in Fig. 1. Upon the addition of oleic acid to an aqueous solution of heavy metal ions, the carboxylic end of oleic acid at an oleic acid/water interface would rapidly capture the metal ion via a complex formation at the interface. Then, the metal-carboxylate complex would diffuse from the interface into the oleic acid layer, resulting in the removal of the aqueous heavy metal ions (Fig. 1a). In addition, the phase transfer of the metal ions could be chemically enhanced by the addition of ethylenediamine (EN) ligand to an aqueous phase (Fig. 1b) since more stable complex is known to be produced with the aid of the EN ligand (Fig. 1c)^29,30^. The formation of an emulsion by mechanical agitation could also improve removal rate and efficiency due to increased interfacial area and concomitant shorter diffusion time (Fig. 1b).

**Figure 2:**
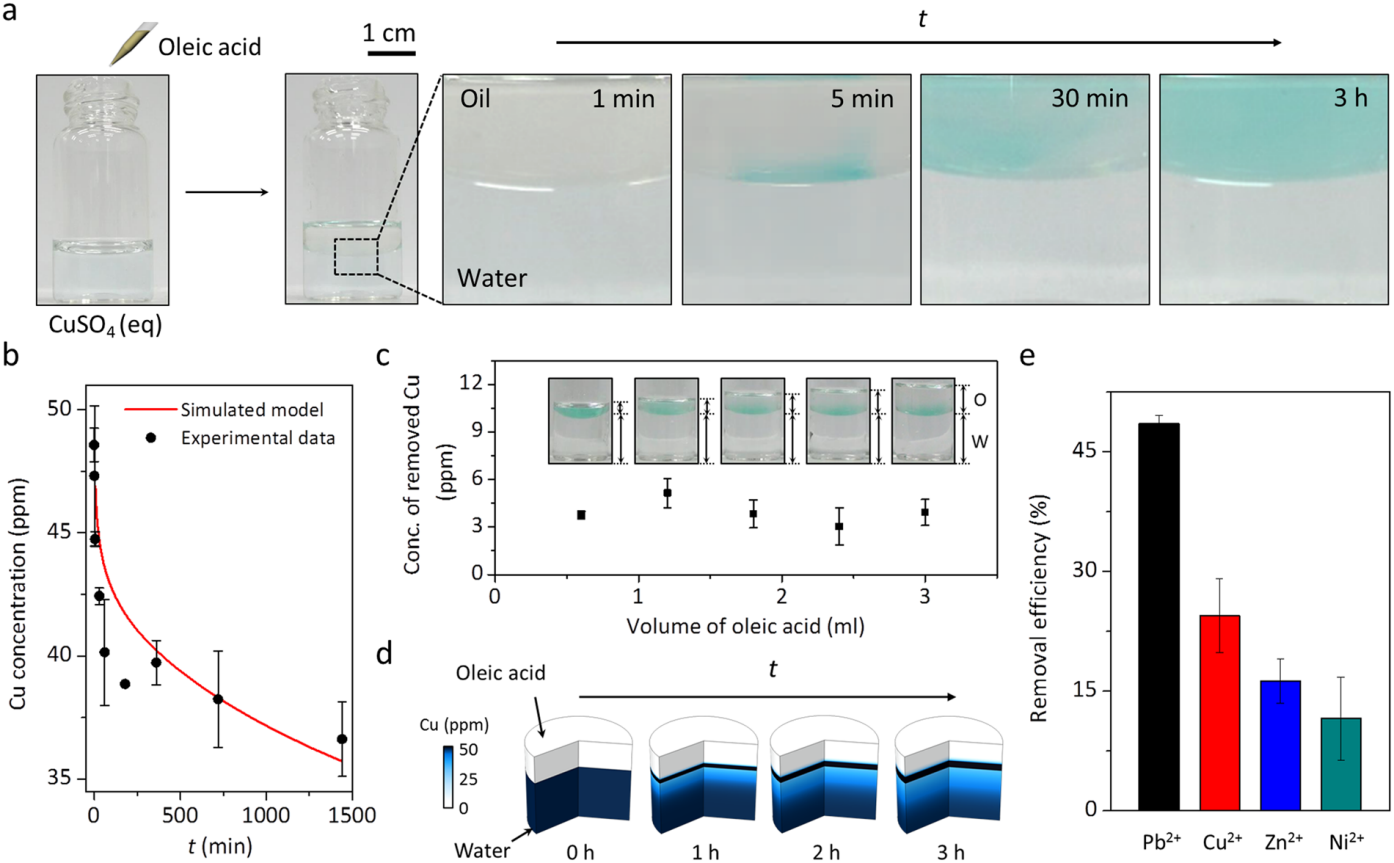
Removal performance for heavy metal ions by oleic acid. (**a**) Photographs showing the phase transfer of Cu^2+^ from water to oleic acid layer over time. (**b**) The concentration of Cu^2+^ in water over time (black dot) and simulated fit (red line). (**c**) Removal capacity for Cu^2+^ by changing the volume of oleic acid. (**d**) Simulated three-dimensional images of Cu^2+^ concentrations at different time. (**e**) Removal efficiencies for various metal ions (Pb^2+^, Cu^2+^, Zn^2+^, and Ni^2+^) after 24 h.

First, removal performance by oleic acid for aqueous Cu^2+^ ion is investigated since Cu^2+^ ion is environmentally and biologically important^31^. After 1.5 ml of oleic acid is added to 3.0 ml of 48 ppm Cu^2+^ solution, transparent oleic acid layer gradually turns blue (Fig. 2a). The result implies that the aqueous Cu^2+^ ion is spontaneously transferred into the oleic acid layer. To quantitatively analyze Cu^2+^ removal by oleic acid, the concentration of the aqueous Cu^2+^ over time is recorded. As shown in Fig. 2b (black dot), the concentration is decreased from 48 to 37 ppm in 24 h. Most of the change in the concentration occurs within the first 1 h, followed by a gradual decrease to 37 ppm.

Red line in Fig. 2b shows the simulation result for the removal kinetics of aqueous Cu^2+^. We assume both diffusion and electrostatic interaction to be mainly responsible for the mass flux of the aqueous Cu^2+^ ^32,33^. Consequently, the total mass flux (*J*_*tot*_) can be expressed as follows:

1 $${J}_{tot}=-D\nabla c-zFuc\nabla \varphi $$

where *D*, z, and *u* are the diffusion constant, the valence, and the ion mobility of Cu^*2+*^ ion. *F* and *ϕ* represent Faraday’s constant and the electrostatic potential, respectively^34^. The law of conservation of mass along with the equation (1) produces the following governing equation:

2 $$\partial c/\partial t=\nabla \cdot (D\nabla c+zFuc\nabla \varphi )$$

The interfacial concentrations of Cu^2+^ (*c*_*Cu*_), oleic acid (*c*_*OA*_), and their complex (*c*_*Cu·OA*_) can be determined from the following equilibrium constant (*K*_*eq*_) (Table S1):

3 $${K}_{{eq}}=\frac{{c}_{{Cu}\cdot {OA}}}{{c}_{{Cu}}\times {c}_{{OA}}}$$

The diameter of a container is 19 mm and the volumes of water and oleic acid are fixed at 3.0 and 1.5 ml, respectively. The simulation in Fig. 2b shows that the concentration of the aqueous Cu^2+^ is drastically decreased at an early stage due to rapid complexation. This tendency is in good agreement with the experimental observation.

Next, the effect of the volume of oleic acid on the removal of Cu^2+^ is examined while maintaining the concentration at 48 ppm. Interestingly, the amounts of Cu^2+^ removed are found to be similar irrespective of the volume of oleic acid (Fig. 2c). The photographs in Fig. 2c (inset) also show that the blue color appears near the interface. The simulated three-dimensional images of Cu^2+^ concentrations over time in Fig. 2d also show significant accumulation of the Cu^2+^ complex around the interface. Both the experiment and simulation suggest that oleic acid near the interface would participate in the complex formation. It is understandable that the poor solubility of the complex in the oleic acid layer could retard the diffusion into the oleic acid layer.

To further test the utility of our method, the removal of other heavy metal ions (Pb^2+^, Zn+, and Ni^2+^) is examined at a similar initial concentration as Cu^2+^. For a comparison, removal efficiency, defined as percentage removal ratio, is calculated. Fig. 2e shows that the removal efficiency at a given concentration follows the order, Pb^2+^ (49%) > Cu^2+^ (24%) > Zn^2+^ (16%) > Ni^2+^ (11%). This result is in good agreement with the order of stability constants for the metal-carboxylate complexes^25^. At lower initial concentrations, the concentration of aqueous Cu^2+^ is reduced to below 1.3 ppm which is the level of the Environmental Protection Agency’s regulation for drinking water (Table S2)^35^.

**Figure 3:**
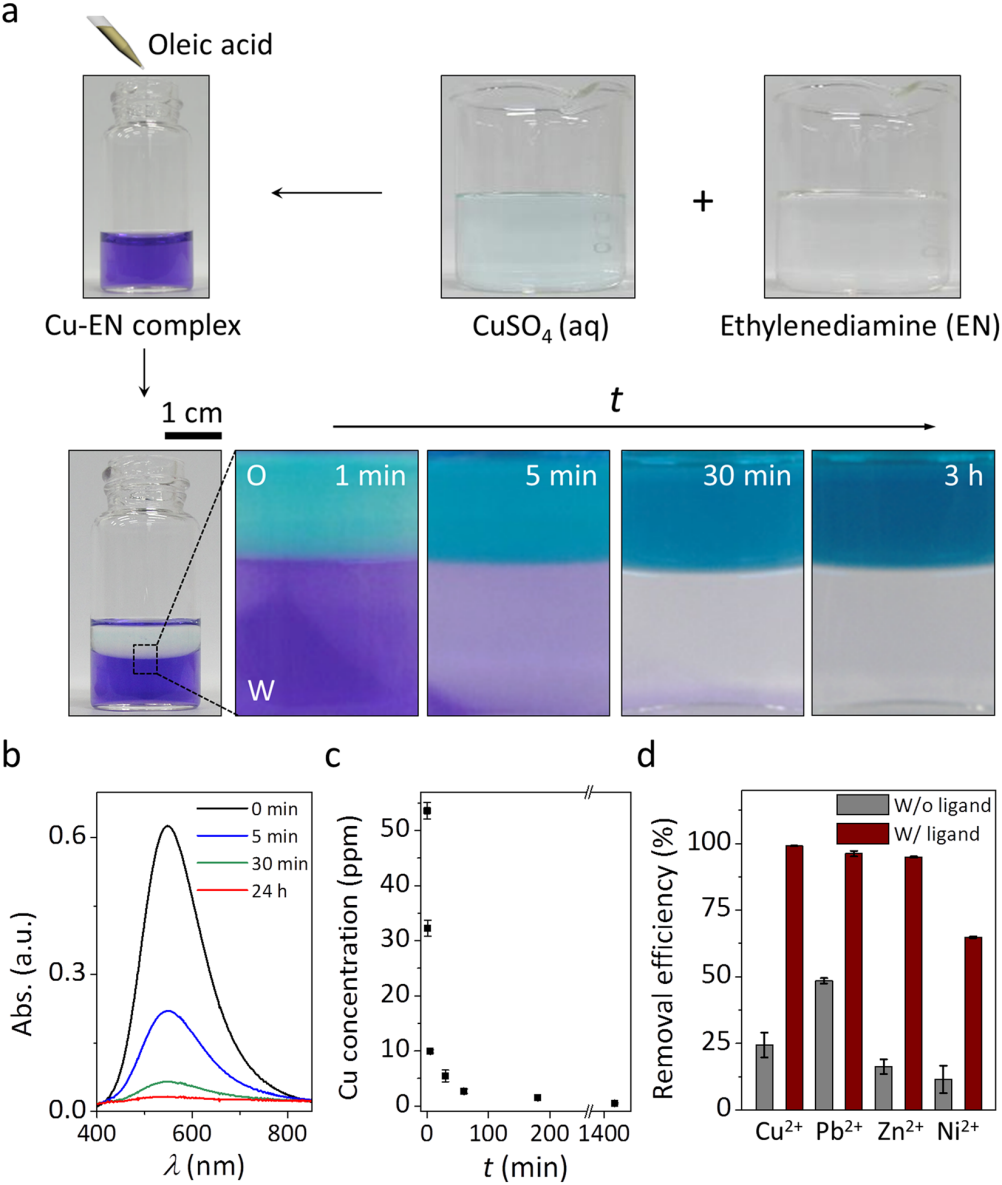
Removal performance for heavy metal ions by oleic acid with the addition of EN into water. (**a**) Photographs showing the phase transfer of Cu^2+^ from water to oleic acid layer over time in the presence of EN. (**b**) Changes of UV-vis spectrum over time after adding oleic acid onto the aqueous Cu^2+^ solution with EN. (**c**) The concentrations of Cu^2+^ in the presence of EN. (**d**) Removal efficiencies for various heavy metal ions (Cu^2+^, Pb^2+^, Zn^2+^, and Ni^2+^) with EN.

Since bidentate EN ligand is known to form a more stable complex with Cu^2+^ than oxalate which contains two carboxylates, the removal of Cu^2+^ by oleic acid with the aid of EN is examined. After the addition of EN into an aqueous Cu^2+^ solution, we observe that the color of the solution immediately turns violet (Fig. 3a top). The violet color would result from the *d*-orbital splitting of the complex between Cu^2+^ and EN^36^. Upon exposure to oleic acid layer, the intense violet color of the aqueous solution disappears while the color of the oleic acid layer changes to greenish blue (Fig. 3a bottom). This color change is further confirmed by using a UV–vis spectrophotometer. As shown in Fig. 3b, the maximum absorbance wavelength of the solution is around 548 nm. The intensity at 548 nm is rapidly attenuated by approximately 75% within 30 min and then gradually decreased until 24 h.

The change in an aqueous Cu^2+^ concentration over time is also measured, as shown in Fig. 3c. Similar to the previous UV–vis result, the concentration in water is decreased from 54 to 0.5 ppm after 24 h. The removal capacity is increased by *ca*. 4 times in comparison with that without EN. The removal rate by oleic acid with the aid of EN becomes faster. For example, the amount of Cu^2+^ removed in the first 30 min is increased by *ca*. 10 times.

The removal efficiencies for other heavy metal ions (Cu^2+^, Pb^2+^, Zn^2+^, and Ni^2+^) by oleic acid with the aid of EN are also evaluated (Fig. 3d). The removal efficiencies for the metal ions are increased by up to *ca*. 6 times relative to those without EN. For example, 95% of Zn^2+^ in the presence of EN is removed within 24 h, whereas only 16% of Zn^2+^ is removed without EN. The removal efficiencies follow the order, Cu^2+^ (99%) > Pb^2+^ (96%) > Zn^2+^ (95%) > Ni^2+^ (65%). Note that the removal efficiency of Cu^2+^ is higher than that of Pb^2+^. This can be contributed to a higher stability constant of Cu^2+^ with EN^37^.

**Figure 4:**
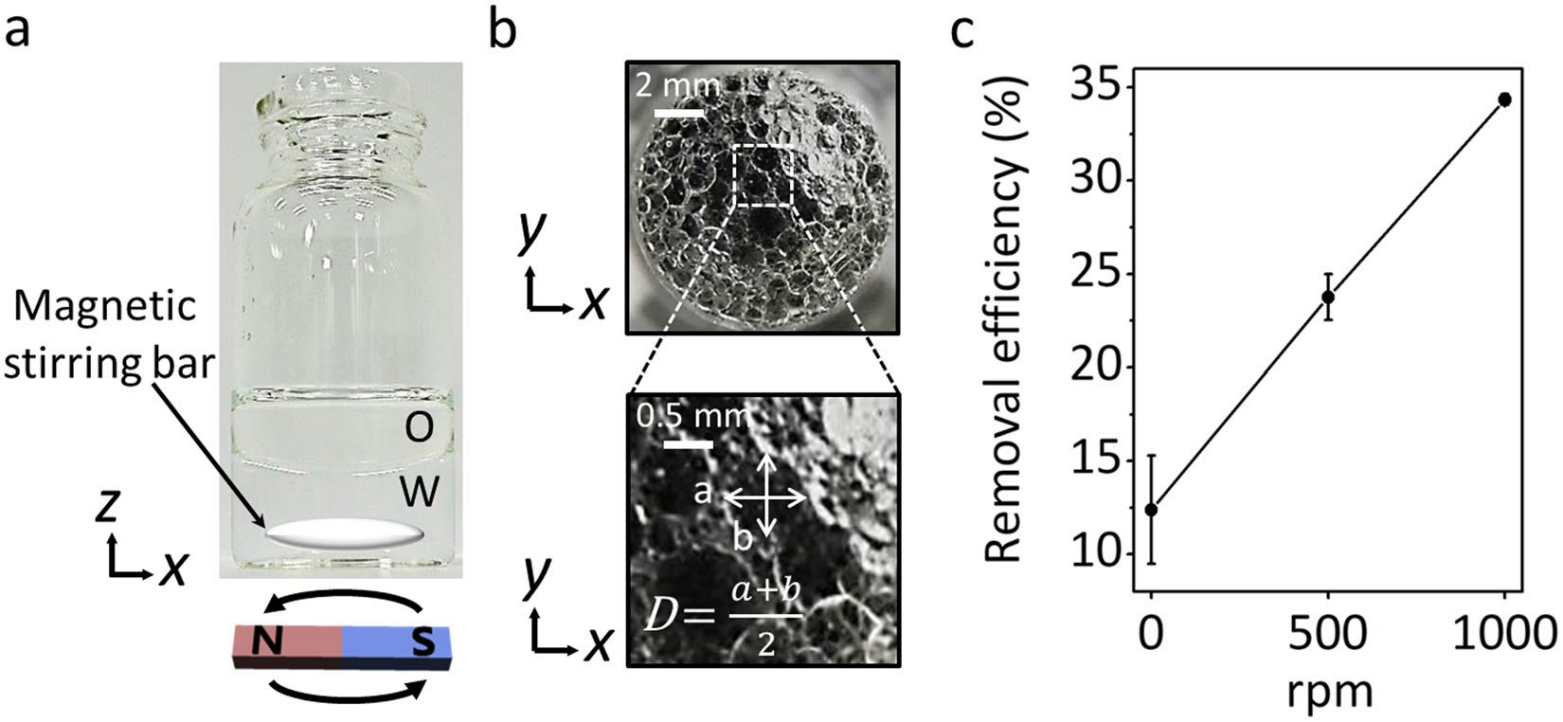
Removal performance for Cu^2+^ by the emulsion of oleic acid. (**a**) Mechanical stirring for the formation of the emulsion. (**b**) Representative photographs of the emulsion at 1000 rpm. (**c**) Removal efficiency in 10 min at different stirring speed.

Since complex formation at an oleic acid/water interface and diffusion across the interface depends on an interfacial area, the effect of an emulsion (*i.e*., oleic acid) on the removal rate and efficiency is addressed. Mechanical stirring for the formation of the emulsion would also reduce the diffusion time of an aqueous metal ion into the interface (Fig. 4a). The photographs in Fig. 4b show the representative emulsion of oleic acid by mechanical agitation. The average emulsion diameter is decreased as the stirring speed is increased (Fig. S1). The removal efficiency at different stirring speeds is shown in Fig. 4c. The removal efficiency is linearly increased from 12 to 35% with increasing the stirring speed from 0 to 1000 rpm.

In this study, we have demonstrated the environment-friendly removal of aqueous heavy metal ions by utilizing spontaneous and selective phase transfer of such ions into oleic acid. This phase transfer is based on metal–carboxylate complexation and diffusion. Upon the addition of oleic acid onto the aqueous metal ion solution, the ions begin to diffuse toward an oleic acid/water interface by either random diffusion or electrostatic interaction. Stable complex between the metal ion and carboxylic end of oleic acid is thus spontaneously formed at the interface, followed by slow diffusion into the oleic acid layer. By using oleic acid alone, it is possible to reduce the concentration of Cu^2+^ below the regulation level by the Environmental Protection Agency for drinking water (*i.e*., 1.3 ppm). Other heavy metal ions such as Pb^2+^, Ni^2+^, and Zn^2+^ can also be removed. Removal performance by oleic acid can also be improved by the EN ligand due to the formation of more stable complex. In the presence of EN, removal efficiency and rate by oleic acid are increased up to *ca*. 6 and 10 times, respectively. In addition, the removal efficiency is increased by *ca*. 3 times by the formation of an emulsion of oleic acid. We believe that our method will have a large impact on a wide variety of environmental applications ranging from environmental monitoring to water purification.

## Methods

### Chemicals and materials

Copper sulfate pentahydrate (99.5%) and nickel nitrate hexahydrate (97%) were obtained from Junsei chemical Co., Ltd. Lead nitrate (99%), ethylenediamine (EN) (99.5%), and oleic acid (technical grade, 90%) were purchased from Aldrich. Zinc chloride (98%) was purchased from Kanto chemical Co., Inc. Deionized (DI) water was used throughout the experiments.

### Preparation of simulated heavy metal ion solutions

Simulated aqueous heavy metal ion solutions were prepared with the 50 ppm CuSO_4_∙5H_2_O, Pb(NO_3_)_2_, Ni(NO_3_)_2_∙6H_2_O, ZnCl_2_ solution with DI water, respectively. In case of adding ligand, these powders were dissolved in 100 ppm of EN solution because it is known that a heavy metal ion generally forms complex with EN of 1:2 stoichiometry^38^.

### Heavy metal ions removal by oleic acid

Heavy metal ions removal experiments were performed in 10 ml vial (1.9 cm diameter). 1.5 ml of oleic acid was added to 3.0 ml of the above simulated solution to examine the removal kinetics and efficiency. To investigate examine the maximum removal capability of oleic acid, we added oleic acid at different volumes (0.6, 1.2, 1.8, 2.4, and 3.0 ml) while fixing of oleic acid to fixed the volume (5.4 ml) of water layer.

### Formation of an emulsion

Mechanical agitation is performed by magnetic stirrer (HS15-26p, Misung Scientific Co., Ltd.) and stirring bar. The maximum rpm of the magnetic stirrer is 1500 rpm. Pictures of emulsion with different stirring speed (0, 500, 1000, 1250, and 1500 rpm) are taken to determine average emulsion sizes statistically. Then we calculated average interfacial area from average emulsion size.

### Determination of heavy metal ion concentrations

The concentrations of aqueous heavy metal ions were analyzed by inductively coupled plasma-optical emission spectrophotometer (ICP-OES, Perkin Elmer Optima 8300). Absorbance spectra were also obtained from UV-vis spectrophotometer (Agilent 8453 G1103A).

### Simulation

In order to quantify the removal kinetics of aqueous Cu^2+^, the local concentration of Cu^2+^ was numerically investigated by solving Fick’s second law of diffusion. The reaction of Cu^2+^ with oleic acid molecules at the oil/water interface was considered with an assumption that the equilibrium is always satisfied at the interface. Diffusivity of Cu^2+^ in water and self-diffusivity of oleic acid itself were set to be 0.7 × 10^−9^ m^2^/s and 0.47 × 10^−10^ m^2^/s, respectively^39–42^. The equilibrium constant without additional ligand was measured from the experimental results and set to be 5.4 × 10^−4^ (Table S1). Numerical simulations are performed by using commercial finite element analysis software (COMSOL Multiphysics 4.4, COMSOL Inc.).

## Electronic supplementary material

Supplementary information
