# Supplementary material for: Spontaneous Phase Transfer-Mediated Selective Removal of Heavy Metal Ions Using Biocompatible Oleic Acid
Source: Sci Rep. 2017 Dec 1;7:16727. doi: 10.1038/s41598-017-17092-9 (PMC5711890; doi:10.1038/s41598-017-17092-9)
Supplement: Supplementary file 1 — Supplementary information [file 41598_2017_17092_MOESM1_ESM.docx]

**Supporting information**

**Spontaneous Phase Transfer-Mediated Selective Removal of Heavy Metal Ions Using Biocompatible Oleic Acid**

Jeehan Chang^a,‡^, Sooyeon Yoo^a,‡^, Wooju Lee^b^, Dongchoul Kim^b^, and Taewook Kang^a,*^

*^a^Department of Chemical and Biomolecular Engineering, Sogang University, Seoul, 04107, Korea*

*^b^Department of Mechanical Engineering, Sogang University, Seoul, 04107, Korea.*

*Corresponding author: E-mail: [twkang@sogang.ac.kr](mailto:twkang@sogang.ac.kr)

‡These authors contributed equally to this work.


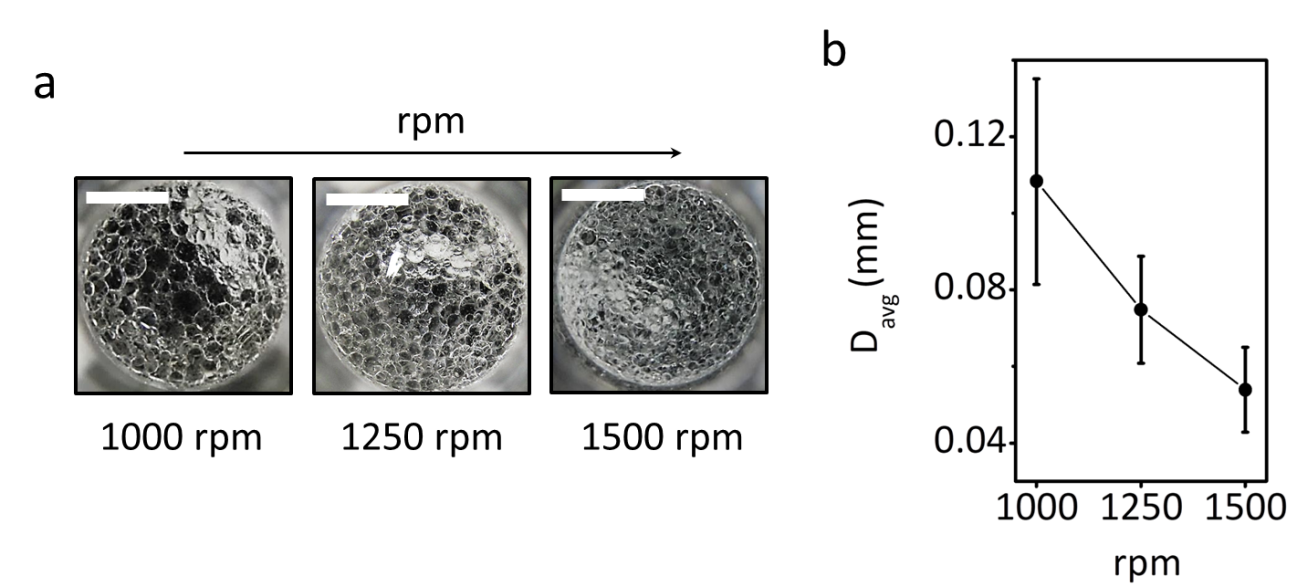


**Figure S1.** Average diameters of emulsion at different stirring speed. (a) Representative photographs of emulsion on top-view at different stirring speed. (b) Average diameter of 30 randomly selected droplets at different stirring speed. Scale bar for photographs is 5 mm.

**Table S1**. Equilibrium constants at different initial concentration of Cu^2+^.

|  | Initial Cu^2+^ concentration (ppm) | Final Cu^2+^ concentration (ppm) | Equilibrium constant (K_eq_$\times$10^4^) | Standard deviation of (K_eq_$\times$10^4^) |
| --- | --- | --- | --- | --- |
| Sample 1 | 21.46 | 9.47 | 8.00 | 0.06 |
| Sample 2 | 47.02 | 32.55 | 2.81 | 0.22 |

We assumed that Cu^2+^ binds to oleic acid with 1:1 stoichiometry and complexation reaction is elementary reaction. Equilibrium constant is determined by equation as follows:

$$K_{eq}=\frac{[M{\cdot OA}^{-}]}{\left[ M^{2+} \right][{OA}^{-}]}=\frac{2(C_{i}-C_{f})}{C_{f}[3170-2\left( C_{i}-C_{f} \right)]}$$

where $C_{i}$,$C_{f}$ are initial, final concentration of Cu^2+^ in aqueous solution, respectively. 3170 means the molarity of pure oleic acid. Since equilibrium constant depends on temperature only, the average value of equilibrium constants of sample 1 and 2 ($K_{eq,avg}=5.4\times{10}^{-4})$ is used for simulation.

**Table S2**. Removal efficiencies in 24 h at low initial concentration of Cu^2+^.

|  | Initial Cu^2+^ concentration (ppm) | Final Cu^2+^ concentration (ppm) | Removal efficiency (%) | Standard deviation |
| --- | --- | --- | --- | --- |
| Sample 3  (w/o EN ligands) | 3.78 | 0.25 | 93.52 | 6.35 |
| Sample 4  (w/ EN ligands) | 4.77 | 0.24 | 95.08 | 4.39 |
